# Comparison of COVID-19 home-testers vs. laboratory-testers in New York State (excluding New York City), November 2021 to April 2022

**DOI:** 10.3389/fpubh.2023.1058644

**Published:** 2023-03-23

**Authors:** Vajeera Dorabawila, Virgile Barnes, Nirmala Ramesh, Rebecca Hoen, Jamie Sommer, Amy Robbins, Byron Backenson, Emily Lutterloh, Dina Hoefer, Eli Rosenberg

**Affiliations:** New York State Department of Health, Albany, NY, United States

**Keywords:** home tests, laboratory tests, COVID-19, race and ethnicity, COVID-19 vaccines, hospitalization, COVID-19 home vs. laboratory testers

## Abstract

**Background:**

Though the use of coronavirus disease 2019 (COVID-19) home testing kits is increasing, individuals who use home tests are not accounted for in publicly reported COVID-19 metrics. As the pandemic and the methods for tracking cases evolve, it is critical to understand who the individuals excluded are, due to their use of home testing kits, relative to those included in the reported metrics.

**Methods:**

Five New York State databases were linked to investigate trends in home-tested COVID-19 cases vs. laboratory-confirmed cases from November 2021 to April 2022. Frequency distributions, multivariate logistic regression adjusted odds ratios (aOR), and 95% confidence intervals (CI) were used to compare the characteristics of the home-tested and laboratory-tested people.

**Results:**

Of the 591,227 confirmed COVID-19 cases interviewed, 71,531 (12%) of them underwent home tests, 515,001 (87%) underwent laboratory tests, and 5,695 (1%) underwent both home tests and laboratory tests during this period. Home-tested COVID-19 cases increased from only 1% in November 2021 to 22% in April 2022. Children aged 5–11 years with an aOR of 3.74 (95% CI: 3.53, 3.96) and adolescents aged 12–17 years with an aOR of 3.24 (95% CI: 3.07, 3.43) were more likely to undergo only home tests compared to adults aged 65 years and above. On the one hand, those who were “boosted” (aOR 1.87, 95% CI: 1.82, 1.93), those in K-12 school settings (aOR 2.33, 95% CI: 2.27, 2.40), or those who were possibly infected by a household member (aOR 1.17, 95% CI: 1.13, 1.22) were more likely to report home testing instead of laboratory testing. On the other hand, individuals who were hospitalized (aOR 0.04, 95% CI: 0.03, 0.06), who had underlying conditions (aOR 0.85, 95% CI: 0.83, 0.87), who were pregnant (aOR 0.76, 95% CI: 0.66, 0.86), and who were Hispanic (aOR 0.50: 95% CI: 0.48, 0.53), Asian (aOR 0.31, 95% CI: 0.28, 0.34), or Black (aOR 0.45, 95% CI: 0.42, 047) were less likely to choose home testing over laboratory testing.

**Conclusion:**

The percentage of individuals with confirmed COVID-19 who used only home testing kits continues to rise. People who used only home testing were less likely to be hospitalized and were those with a lower likelihood of developing a severe disease given factors such as age, vaccination status, and underlying conditions. Thus, the official COVID-19 metrics primarily reflected individuals with severe illness or the potential for severe illness. There may be racial and ethnic differences in the use of home testing vs. laboratory testing.

## 1. Introduction

The coronavirus disease 2019 (COVID-19) pandemic is ongoing, and there is a continued need to monitor its trends. As of September 21, 2022, there were almost 6 million COVID-19 cases and 400,000 reinfections in New York State (NYS). The prevalence of at-home COVID-19 antigen tests (home tests) has rapidly increased across the United States since the emergence of the B.1.2.529 (Omicron) variant in late 2021 (1–8). During the surge of cases with the Omicron variant in late 2021 and January 2022, there was a shortage of laboratory-based diagnostic nucleic acid amplification testing (laboratory-based tests), resulting in an increase in the use of home tests (6). A recent national study reported that the usage of at-home tests by those with COVID-19-like illness increased from ~5.7% in the delta-variant dominant period (August 23, 2021–December 11, 2021) to 20.1% in the omicron-variant dominant period (December 19, 2021–March 12, 2022) (9). Another study reported an increasing trend in home-test use and found that, the week ending on January 8, 2022, when testing volume peaked, had a positivity rate of 17.3 for home tests and 29.1 for laboratory-based tests (10). While the two test types differ in sensitivity and specificity, home tests are easily accessible, offer greater privacy in testing, and provide rapid results compared to laboratory-based tests (2). Simultaneously, the increase in the accessibility of home tests was driven by free test kits provided by schools, the state, and the federal government and increased availability of home test kits in pharmacies and retail venues (11).

Official reports and other analyses were primarily based on laboratory-based testing, as home tests were not typically included in data systems that track COVID-19 cases and rates. Consequently, there was a dearth of information on home test usage and users; the research available on this topic is limited to surveys that have a higher risk of response bias and were conducted on adults (12, 13). Given this fact, there is a critical need to better understand individuals, including children, who use and report home tests, as well as the trends in home testing. Such an analysis will help identify any potential bias in the reports based solely on laboratory-based testing.

This study explored several questions associated with home tests in NYS, excluding New York City (NYC). First, the trends in at-home tests that were voluntarily reported to local health departments (by phone, email, and online), which were then added to the public health surveillance system were compared with laboratory-based testing. Second, this study aimed to understand the proportion of individuals who use at-home and laboratory-based tests. Third, this study also aimed to compare people who reported having taken at-home tests to those who reported having taken laboratory-based tests based on the participants' demographics, K-12 school-based attendance or workplace settings, vaccination status, the severity of disease, symptoms, and knowledge of the source of infection.

## 2. Materials and methods

### 2.1. Materials

This study used five NYS databases that were linked together (14, 15). The primary database for COVID-19 case investigation in NYS, outside of NYC, is the Communicable Disease Case Management System (CDCMS). The Electronic Clinical Laboratory Reporting System (ECLRS) contains all reportable (positive and negative) COVID-19 test results [nucleic acid amplification test (NAAT) or antigen] in NYS. However, home tests are not reported to the ECLRS. Applicable information on all positive laboratory tests is automatically transmitted from the ECLRS to the CDCMS. A new case in the CDCMS is created if the positive specimen collection date is >90 days from the specimen collection date of the first positive result or the most recent case specimen collection date (16). Local Health Departments (LHDs) can manually enter cases with positive tests (NAAT or antigen) not reported *via* the ECLRS. These cases could be at-home tests reported to LHDs or laboratory reports from another jurisdiction. LHDs collect data on positive home tests *via* multiple mechanisms. Some mechanisms involve web portals utilized by the public to enter results while others involve receiving data from schools and manual mechanisms such as emails. These are then uploaded to the CDCMS and utilized for case management.

To determine vaccine status, cases in the CDCMS were linked to a combined database that contained the Citywide Immunization Registry (CIR) and the NYS Immunization Information System (NYSIIS) COVID-19 vaccination data for the residents of New York City and the rest of NYS, respectively. These databases were linked using deterministic algorithms that matched individuals based on their name and date of birth (DOB). Finally, the Health Electronic Response Data System (HERDS) is a statewide daily electronic survey conducted to collect information about patients with COVID-19 who were admitted to inpatient facilities. HERDS-reported hospital admissions were linked based on patients' name initials, sex, date of birth, and ZIP code.

The analysis period started, from November 1, 2021, when the home test entry to the CDCMS became more systematic, and was limited to cases with completed interviews that were conducted by case investigators. End of the period was April 30, 2022. The main outcome of this study was to determine the occurrence of COVID-19 cases. A COVID-19 case (or simply “case”) was identified as a new positive result for the SARS-CoV-2 NAAT or antigen test (including at-home tests) that occurred more than 90 days from the first or previous positive result (16). The created cases that were entered manually into the system were excluded if the type of test (laboratory-based or at-home tests) was unclear. Since only limited counties in NYS investigated at-home tests in the CDCMS, the analysis was restricted to 33 counties that reported routinely uploading at-home test results to the CDCMS.

This study presents trends in reported testing for three mutually exclusive groups based on how cases were reported as testing positive. The testing groups were home-test-only, laboratory-test-only, and both at-home and laboratory-based tests (both tests). The cases were classified as home-test-only vs. laboratory-based test-only using the test types available in the CDCMS. Home-test cases were linked to the ECLRS to identify people with laboratory tests within 7 days (before, same day, or after) of the at-home test, which was further classified as both tests. The status of being fully vaccinated was defined as an individual having completed the primary vaccination series and having waited at least 14 days thereafter. The status of being “boosted” was defined as having received a booster shot and having waited at least 1 day after the booster receipt.

### 2.2. Methods

The analysis provided descriptive statistics (demographics, geographic locations, K-12 school-based setting, vaccination status, symptoms, underlying conditions, and known exposure locations) for each of the three case testing groups. The school setting was based on those who responded that “within the 2 weeks prior to symptom onset/collection date”, they did “visit/attend” a “school/university/childcare center”. Those who selected “School (Prek-12)” as the type of school or summer camp they visited were classified as “K-12 school-based”. Then, they were offered a list of roles in school (student, staff, faculty, teacher, volunteer, or visitor); this information was utilized to determine the sub-categories in “K-12 school-based” settings. Exposure type was based on the question, “in the past 14 days, have you been in contact with a COVID-19 Case?”, and if “yes”, the type of exposure was selected by the participants from an offered list. Based on the list of options offered in the CDCMS, the symptoms were classified into the following categories: (a) gastrointestinal (abdominal pain, dehydration, diarrhea, vomiting, and nausea); (b) back and muscle pain; (c) cold symptoms (chills, cough, fatigue, fever, headache, runny nose, and sore throat); (d) cardiac, respiratory, and rigor (chest pain, difficulty breathing, shortness of breath, wheezing, rigor, and seizure); and (e) smell and taste. Exposure type and symptoms are not mutually exclusive categories. A case can be classified into multiple exposure types and symptom categories given that a person has multiple instances of exposure and/or symptoms. The severity of the disease was measured as the number of hospitalizations (reported to HERDS) within 14 days of the specimen collection date (those within 7 days were also examined).

The study employed multivariate logistic regression to estimate adjusted odds ratios (aOR) with 95% confidence intervals (CIs). Specifically, the study calculated aORs for the following comparisons: (a) trends adjusted for the month, county, and age category for the home-test-only group compared to the laboratory-test-only group and the home-test-only group compared to the both tests group; and (b) home-test-only vs. laboratory-test-only were further adjusted for race/ethnicity, setting, underlying conditions, pregnancy, exposure source, and symptoms. To account for geographic differences and varying practices related to home test usage, we included the county variable in the analysis. Adjusting for the case reported month accounts for temporal variation in home test volume, especially with evolving variants. To categorize the study population by age, broad age categories were used: 0–4 years, 5–11 years, 12–17 years, 18–49 years, and 50–64 years, with above 64 years serving as the reference category. The reference category for race/ethnicity was white, while the reference category for gender was female gender. This study also included participants from K-12 schools, such as students, staff, faculty, and volunteers, with a reference category of individuals who did not belong to these groups (non-school based). All statistical analyses were conducted using Statistical Analysis Software (SAS 9.4). The New York State Department of Health institutional's review board (IRB) determined this surveillance activity to be necessary for public health purposes and thus it did not require IRB review.

## 3. Results

### 3.1. Trend of home tests

**Figure 1:**
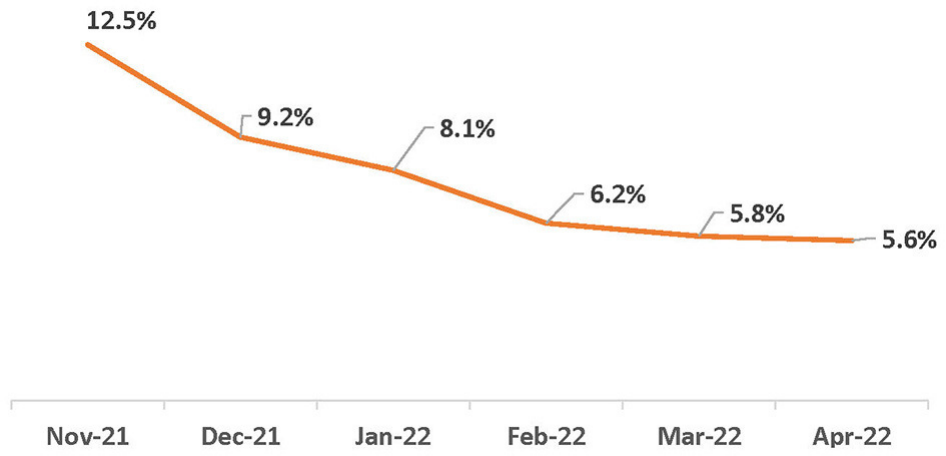
Trend in both-tests (home-test and laboratory confirmed test) as % of any with home tests (home-tests-only and both-tests): November 2021–April 2022.

**Figure 2:**
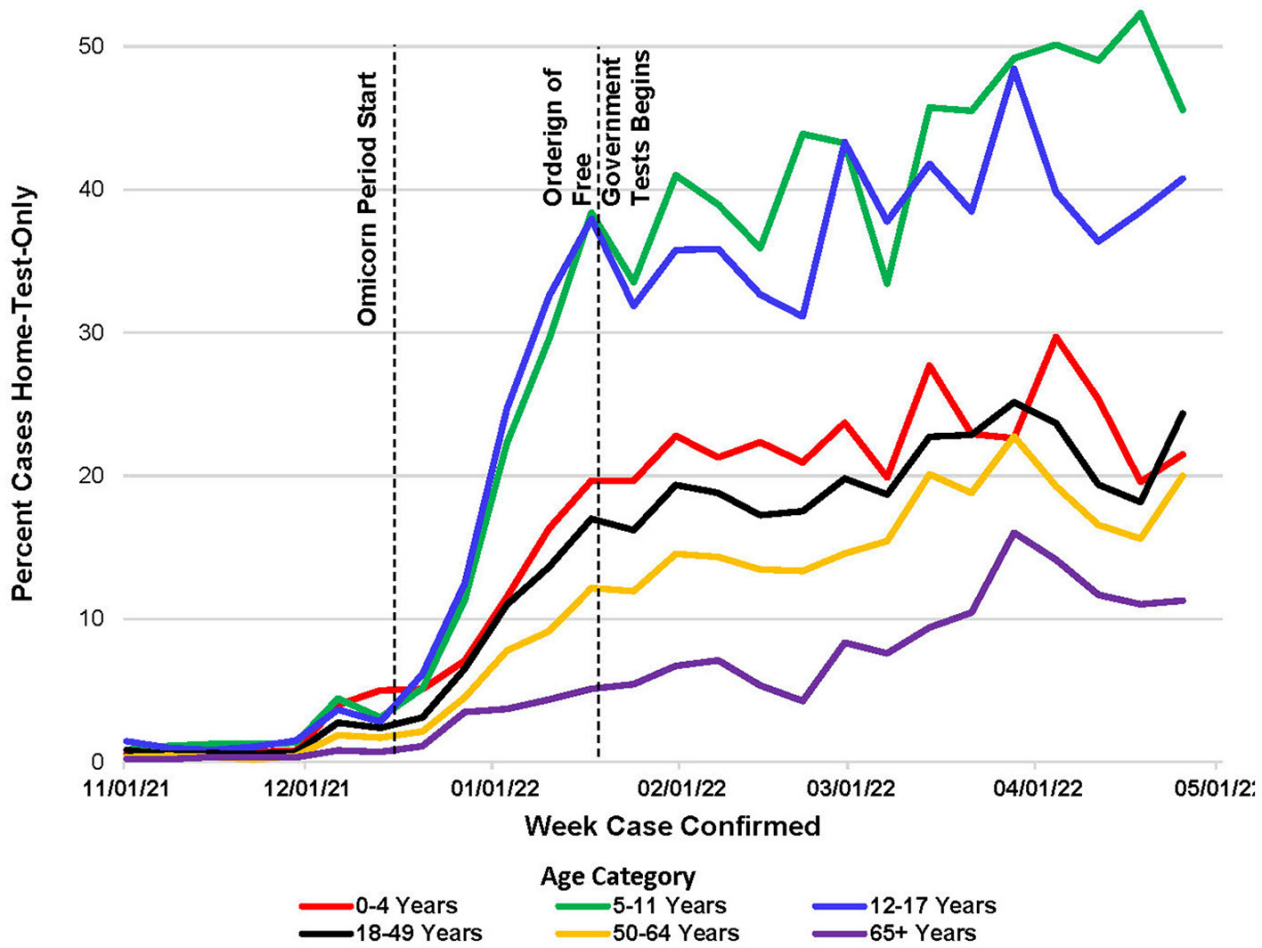
Percent of cases *via* home-tests-only (of all cases), by week and age category, November 2021–April 2022.

From November 2021 to April 2022, there were 592,227 confirmed COVID-19 cases (at-home or laboratory-based) with complete interviews from 33 local health departments (LHDs) that actively investigated at-home tests in the CDCMS. Of the confirmed cases, 71,699 (12%) of them were attributed to home-test-only usage, 515,001 (87%) were due to laboratory-test-only usage, and 5,527 (1.0%) received both types of tests (Table 1). The prevalence of home-test-only cases increased (Table 1; Figure 1) from 0.7% in November 2021 to 21.6% in April 2022. With an increase in the prevalence of home-test-only usage, individuals who received both tests decreased from 12.5% in November 2022 to 4.3% in April 2022. School-aged children (aged 5–17 years) had a higher percentage of home-test-only usage (Figure 2). Of the people who received both tests, 28.1% of them underwent a laboratory-based test on the same day as the home test, while the majority underwent the laboratory-based tests within 7 days after the home test (67.6%).

**Table 1:** Trend in test type: November 2021–April 2022.

	**Home-test-only**	**Laboratory-test-only**	**Both-tests**	**Total**
	***N*** **(%)**	***N*** **(%)**	***N*** **(%)**	***N*** **(%)**
**Total**	**71,531 (12.1)**	**515,001 (87.0)**	**5,695 (1.0)**	**592,227**
November-21	547 (0.7)	78,917 (99.2)	78 (0.1)	79,542
December-21	4,768 (3.5)	131,416 (96.2)	485 (0.4)	136,669
January-22	39,806 (15.8)	208,416 (82.8)	3,496 (1.4)	251,718
February-22	8,331 (19.5)	33,866 (79.2)	552 (1.3)	42,749
March-22	5,654 (23.6)	17,974 (75.0)	346 (1.4)	23,974
April-22	12,425 (21.6)	44,412 (77.1)	738 (1.3)	57,575

People who underwent laboratory testing within 7 days (before, same day, or after) of the at-home test were classified as both-tests.

### 3.2. Comparison of home-test-only usage vs. laboratory-test-only usage

The demographic characteristics are available in Table 2. Among the groups who were tested, the unvaccinated cases comprised a higher percentage in the laboratory-test-only group (46.5%) compared to the both-tests group (32.0%) and the home-test-only group (38.7%). Conversely, people with booster shots represented a higher percentage in the at-home test group (23.6 and 26.4% for the home-test-only group and the both tests group, respectively) compared to the laboratory-test-only group (15.3%). Among the cases reported, the highest percentage of individuals reporting working, attending, or volunteering at a K-12 school was observed in the at-home-test-only group (41.8% for home-test-only usage and 26.5% for both tests) compared to the laboratory-test-only group (19.8%). The prevalence of gastrointestinal symptoms (13.6 vs. 14.7%), cardiac/respiratory/rigor symptoms (7.7 vs. 11.1%), and smell/taste symptoms (6.3 vs. 13.0%), as well as underlying conditions (15.0 vs. 21.4%), were lower for home-test-only cases compared to the laboratory-test-only cases.

**Table 2:** Profile of the laboratory-test-only group, the home-test-only group, and the both-tests group.

	**Home-test-only**	**Lab-test-only**	**Both-tests**
**Total**	**71,531**	**515,001**	**5,695**
	***N*** **(%)**	***N*** **(%)**	***N*** **(%)**
**Age group**			
0–4 years	3,854 (5.4)	24,810 (4.8)	158 (2.8)
5–11 years	12,861 (18.0)	45,863 (8.9)	512 (9.0)
12–17 years	11,668 (16.3)	40,619 (7.9)	565 (9.9)
18–49 years	31,172 (43.6)	252,551 (49.0)	3,363 (59.1)
50–64 years	9,118 (12.7)	97,796 (19.0)	893 (15.7)
65+ years	2,858 (4.0)	53,362 (10.4)	204 (3.6)
**Gender**			
Female	37,175 (52.0)	275,142 (53.4)	3,039 (53.4)
Male	29,302 (41.0)	236,690 (46.0)	2,193 (38.5)
Non-binary	95 (0.1)	93 (0.0)	8 (0.1)
Other	61 (0.1)	715 (0.1)	6 (0.1)
Missing	4,898 (6.8)	2,361 (0.5)	449 (7.9)
**Race/ethnicity**			
Hispanic	2,741 (3.8)	38,266 (7.4)	230 (4.0)
Asian	565 (0.8)	9,021 (1.8)	68 (1.2)
Black	2,421 (3.4)	28,468 (5.5)	266 (4.7)
White	45,525 (63.6)	303,682 (59.0)	3,385 (59.4)
Native American	463 (0.6)	2,163 (0.4)	38 (0.7)
Pacific Islander	52 (0.1)	413 (0.1)	6 (0.1)
Other	2,101 (2.9)	21,998 (4.3)	141 (2.5)
Missing	20,404 (28.5)	149,256 (29.0)	1,791 (31.4)
**Vaccination status**			
Unvaccinated	27,706 (38.7)	239,389 (46.5)	1,821 (32.0)
Partial	2,349 (3.3)	17,059 (3.3)	197 (3.5)
Primary series only	24,626 (34.4)	179,564 (34.9)	2,173 (38.2)
Boosted	16,850 (23.6)	78,989 (15.3)	1,504 (26.4)
**K-12 school vs. non-school**			
Non-school	41,655 (58.2)	413,205 (80.2)	4,183 (73.5)
School (K-12)	29,876 (41.8)	101,796 (19.8)	1,512 (26.5)
Faculty/teacher	4,437 (6.2)	13,080 (2.5)	342 (6.0)
Student	22,442 (31.4)	77,308 (15.0)	929 (16.3)
Staff (non-teacher)	2,799 (3.9)	10,339 (2.0)	227 (4.0)
Volunteer/visitor	198 (0.3)	1,069 (0.2)	14 (0.2)
**Hospitalization**			
Within 7 days	5 (0.0)	13,428 (2.6)	60 (1.1)
Within 14 days	44 (0.1)	15,063 (2.9)	79 (1.4)
**Symptoms**			
Any symptoms	62,661 (87.6)	418,744 (81.3)	4,471 (78.5)
Gastrointestinal	9,737 (13.6)	75,775 (14.7)	826 (14.5)
Back and muscle pain	17,942 (25.1)	133,072 (25.8)	1,560 (27.4)
Cold symptoms	55,661 (77.8)	353,745 (68.7)	4,031 (70.8)
Cardiac, respiratory, and rigor	5,527 (7.7)	56,997 (11.1)	591 (10.4)
Smell and taste	4,511 (6.3)	66,991 (13.0)	466 (8.2)
**Underlying conditions**	10,707 (15)	110,332 (21.4)	983 (17.3)
**Pregnant**	304 (0.4)	3,324 (0.6)	46 (0.8)
**Exposure source known**	7,868 (11.0)	90,742 (17.6)	605 (10.6)
**Known exposure type**			
Congregate housing	18 (0.0)	385 (0.1)	2 (0.0)
Day care/school	741 (1.0)	4,854 (0.9)	62 (1.1)
Place of employment	499 (0.7)	8,569 (1.7)	65 (1.1)
Healthcare facility	21 (0.0)	607 (0.1)	7 (0.1)
Living in same household	4,562 (6.4)	46,836 (9.1)	284 (5.0)
At home, from visitor to home	442 (0.6)	6,576 (1.3)	45 (0.8)
Long-term care facility	15 (0.0)	479 (0.1)	3 (0.1)
Political rally/gathering	3 (0.0)	18 (0.0)	–(0.0)
Religious gathering	19 (0.0)	216 (0.0)	–(0.0)
Sports event	53 (0.1)	312 (0.1)	2 (0.0)
Social event	240 (0.3)	4,672 (0.9)	25 (0.4)
Travel	21 (0.0)	325 (0.1)	2 (0.0)
Summer camp	0 (0.0)	5 (0.0)	0 (0.0)
Other	505 (0.7)	7,237 (1.4)	64 (1.1)
Unknown	82 (0.1)	1,265 (0.2)	8 (0.1)

People who underwent laboratory testing within 7 days (before, same day, or after) of the at-home test were classified as both-tests.

### 3.3. Adjusted trends: Home-test-only usage vs. laboratory-test-only usage

**Figure 3:**
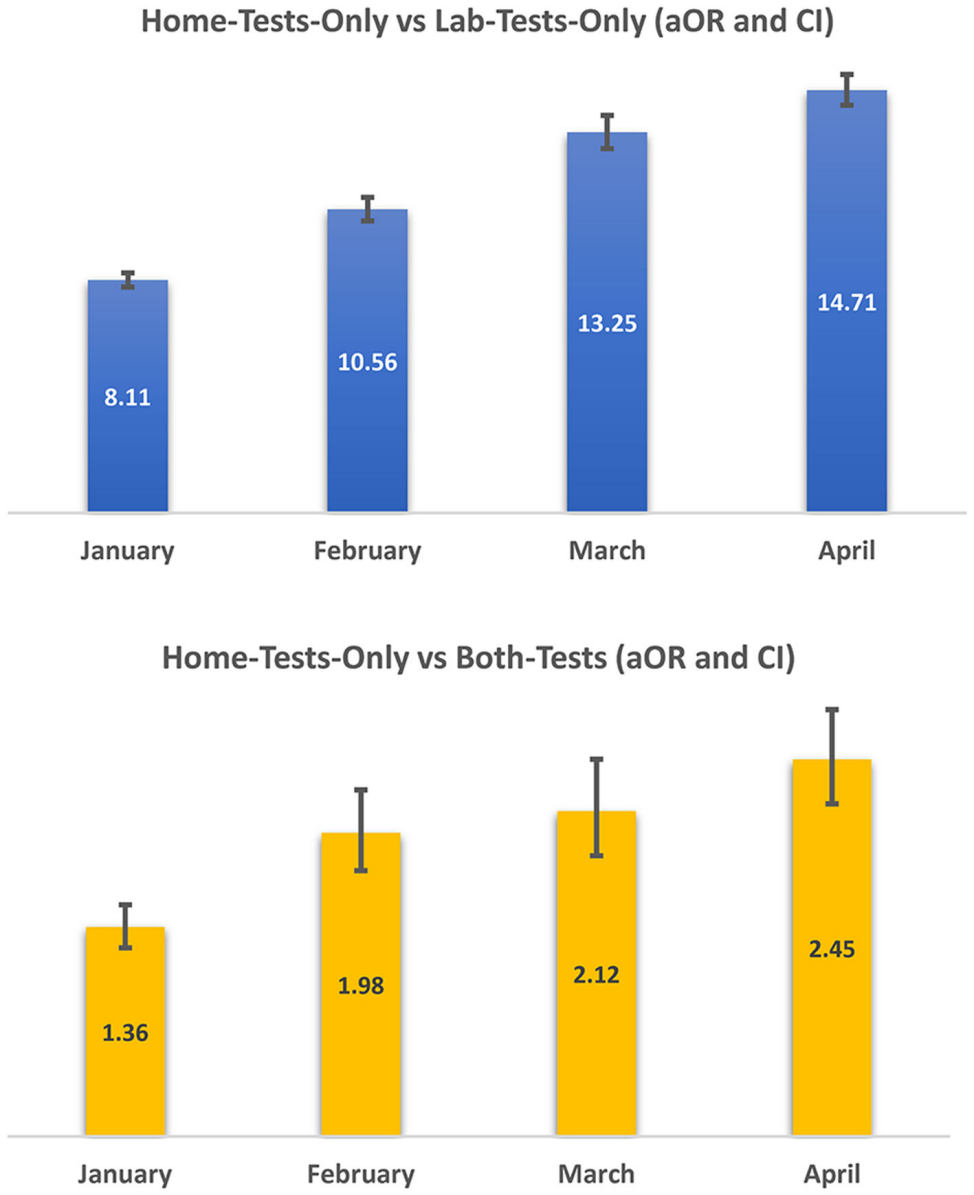
Trend in test type for COVID-19 cases January–April, 2022 relative to November/December 2021: adjusted odds ratios (aOR) and confidence intervals (Cl)^a^. ^a^Adjusted odds ratios (aORs) are adjusted for age and county. As demonstrated in Table 1 and Figure 2, the number of home-tests was small in November/December 2021. Therefore, in estimating aORs, of trends above, combined November/December 2021 case numbers were used as the reference category.

Figure 3 displays the aOR and CI trends for home-test-only usage vs. laboratory-tests-only usage and home-test-only usage vs. both tests while adjusting for age and county. Due to the small number of cases in November 2021, the month of November was combined with the month of December, and the figure provides the monthly trend relative to November and December 2021. The increasing trend in the home-test-only group was consistent. The aOR of the home-test-only group vs. the laboratory-test-only group went from 8.11 (95% CI: 7.86, 8.35) in January 2022 to 14.71 (95% CI: 14.19, 15.26) in April 2022 compared to November/December 2021. There was a concurrent increase in home-test-only usage compared to both tests (a person with an at-home test having a laboratory-based test within 7 days of an at-home test), increasing from 1.36 (95% CI: 1.23, 1.31) in January 2022 to 2.45 (95%CI: 2.17, 2.78) in April 2022 compared to November/December 2021.

### 3.4. Adjusted odds ratios: Comparison of home-test-only usage vs. laboratory-test-only usage

**Figure 4:**
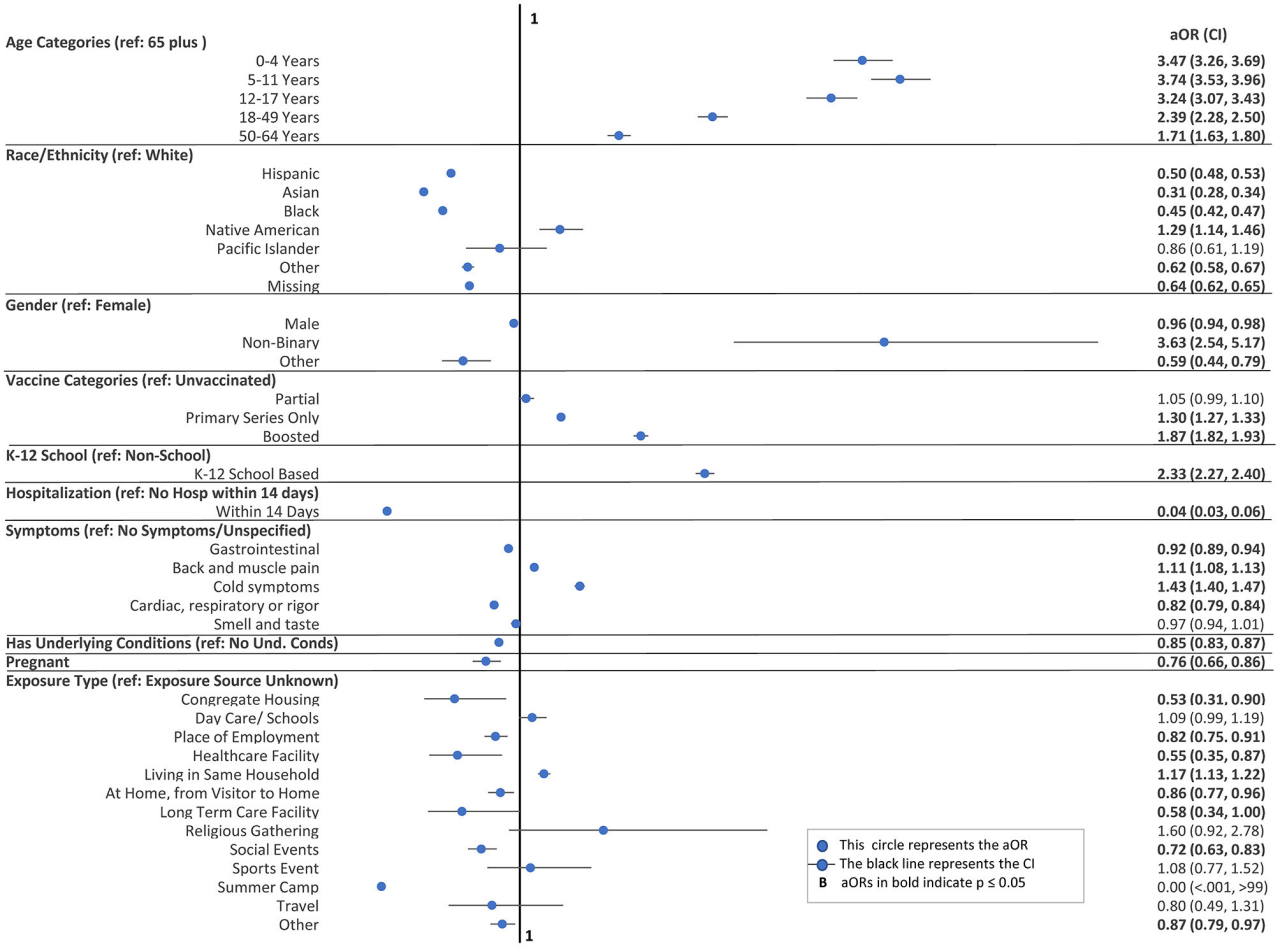
Multivariate logistic regression: adjusted odds ratios (aOR) and confidence intervals (Cl) for home-tests-only vs. laboratory-test-only^b^. ^b^The following variables adjusted in the model are not displayed above: month, county, gender missing, and some exposure sources (political rally/gathering, summer camp, and not reported).

Figure 4 demonstrates that once adjusted for the month, county, and other variables displayed, the differences between home-test-only usage vs. laboratory-test-only usage (observed with descriptive statistics in Table 2) persisted and were statistically significant. Younger people were more likely (aOR range 2.39 to 3.74; 95% CI range 2.28–3.96 for those under 50 years of age) to utilize home tests only compared to those aged 65 years or older. People with boosted shots were most likely to report using home tests (aOR 1.87, 95% CI: 1.82, 1.93) than laboratory tests, followed by those with only a primary series (aOR 1.30, 95% CI: 1.27, 1.33) with unvaccinated people as the reference category. This implies that unvaccinated individuals were more likely to use only laboratory tests. Moreover, the analysis did not find a statistically significant difference for partially vaccinated individuals.

Those in K-12 settings were more likely to use home tests, while those in non-K-12 settings were more likely to use laboratory tests. The aOR for the home-test-only group was 2.33 (95% CI: 2.27, 2.40) relative to the laboratory-test-only group for those in K-12 settings. When we restricted the analysis to the school-aged (5–17 year old) population, the aOR declined to 1.55 (95% CI: 95%CI: 1.46, 1.65) (Supplementary Table A.2). In contrast, when the analysis was restricted to adults (>18 years) only, the aOR increased to 2.85 (95% CI: 2.75, 2.95) (Supplementary Table A.3).

Laboratory-tests-only capture severe disease, as measured by the rate of hospitalizations, certain symptoms, and cases with underlying conditions (Figure 4). The prevalence of hospitalization within 14 days was much less frequent among those who used only home tests rather than laboratory tests, with only 44 home-test-only cases (aOR 0.04, 95% CI: 0.03, 0.06) being hospitalized. The results for hospitalizations within 7 days, with only five home-test-only cases requiring hospitalization, were consistent. People with underlying conditions (aOR 0.85, 95% CI: 0.83, 0.87) or who were pregnant (aOR 0.76, 95% CI: 0.66, 0.86) were less likely to undergo only home tests, as were those with gastrointestinal (aOR 0.92, 95% CI: 0.89, 0.94) and cardiac, respiratory, and rigor (aOR 0.82, 95% CI: 0.79, 0.84) symptoms. People with certain symptom groups (controlled for other symptoms) were more likely to undergo only home tests, with 1.11 (95% CI: 1.08, 1.13) for back and muscle pain and 1.43 (95% CI: 1.40, 1.47) for cold symptoms relative to those without any symptoms reported.

For cases with a known exposure source, the source of exposure was associated with the testing group. If the known exposure source was from a household member (aOR 1.17, 95% CI: 1.13, 1.22), then people were more likely to use only home tests. In contrast, those with known exposure to a case in congregate housing (aOR 0.53, 95% CI: 0.31, 0.90), a place of employment (aOR 0.82, 95% CI: 0.75, 0.91), a healthcare facility (aOR 0.55, 95% CI: 0.35, 0.87), a long-term care facility (aOR 0.58, 95% CI: 0.34, 1.00), or a social event (aOR 0.72, 95% CI: 0.63, 0.83) were less likely to use only home tests.

## 4. Discussion

By linking several databases, this study provides valuable insight into trends in home testing and the people excluded from official laboratory-confirmed test-based metrics in New York State (excluding New York City). The strengths of this study include analysis of tests during the Omicron-variant-dominant period with widespread home testing coverage and population-based large sample sizes using case investigation data collected by trained interviewers in contrast to survey data impacted by response bias. This demonstrated an increase in home testing usage during the period covered and indicated that, at only one percent, only a small portion used both tests. This study fills a gap in the literature by examining the association between testing groups and vaccination choices by utilizing vaccine registries (in contrast to self-reporting) to obtain vaccine histories for all three testing groups. Overall trend in home-testing vs laboratory-based testing as well as race, ethnicity and vaccination status differences between testing groups observed in this study is consistent with that reported in survey-based studies (9, 12, 13, 17) and could be compared with each other. This study demonstrates that certain populations are more likely to use only home tests. Specifically, those at a lower risk of severe diseases, such as those who are boosted, those with certain symptom categories, younger age groups, people not hospitalized, and those without underlying conditions or special medical needs, are more likely to use only home tests.

Racial differences in home testing, with a higher likelihood for white people and a lower likelihood for Black, Asian, Native American, and Hispanic people, may not only reflect testing behavior. The reasons could be accessibility, knowledge, or economic differences in testing (6, 18), particularly given that COVID-19 has disproportionately impacted communities of color (19–24). While data indicate that non-binary people are more likely to utilize only home tests, it is difficult to have conclusive evidence, given the higher percentage of gender missing among home testers (6.9% for home-test-only users, 0.5% for laboratory test-only users) as well as the small number of people who had identified as non-binary (95 who use home tests and 93 who use laboratory tests).

The age-related differences in home testing observed in our study are consistent with those reported in other studies on adults (9). The comparison of adults to the school-age population provides additional insight, as the higher likelihood of home testing observed for the school-aged population is consistent with that observed in a recent study (25). This trend may be driven by the sharp rise in Omicron-variant cases and may have continued with widespread availability of test kits in schools, in schools' requirements for negative test results in case of symptoms, and concerns about transmission risk to other household members (11, 26, 27). Moreover, when we restricted the analysis to only adults, the higher likelihood of home-test-only usage for K-12 school-based people indicated that both age and school setting could impact test choice.

The likelihood of the reported home tests based on the vaccination status may have been impacted by four factors. First, employment testing requirements may have played a role, particularly for unvaccinated individuals (28). Second, if unvaccinated people tested positive, they may have been more likely to opt for laboratory-confirmed testing due to the potential for a more severe disease when presenting with symptoms. Third, given the reverse likelihood of severe disease for boosted, they may opt for home testing. It has been demonstrated that vaccination reduces severity (29, 30), and further analysis on vaccination, boosters, and severity is warranted. Fourth, people who are more conscientious about being vaccinated may be more likely to test at home and report.

People hospitalized with certain symptoms (cardiac, respiratory, or rigor) and underlying conditions are more likely to have severe disease or the potential for developing severe disease. These groups are more likely to choose laboratory tests over home tests. This finding indicates that publicly reported metrics that rely on laboratory tests capture a high proportion of people with severe disease or those with the potential for developing severe disease.

The association between testing types for those with a known exposure source was unsurprising. Healthcare facilities, congregate housing, and long-term care facilities are all congregate care settings where laboratory-testing confirmation may be desired by employers and administrators for both staff and residents. While the risk of transmission from household members was higher (12–24, 26, 28, 31–33), the perception that a home test was sufficient may result in those exposed by a household member opting for only home tests. Both an increased availability of free home tests *via* schools and similar perceptions may lead to those exposed in a daycare or school using only home tests. In addition, there may be costs associated with laboratory tests (2).

There are several limitations to this study. First, the analysis was restricted to LHDs who were willing and able to accept home-test reports. Among the LHDs who were willing and able to accept, home tests were from those who voluntarily reported, while the comparison group included laboratory-based testing which required reporting to ECLRS. The conclusions of this study may be influenced by people's willingness to report the use of home-based tests. One would expect people with severe forms of the disease to seek medical care, and healthcare facilities would require laboratory confirmations, which would be included in the study. For example, according to a recent survey, laboratory-confirmed tests had an estimated test positivity rate of 14.1% for COVID-19 infection, whereas the rate was 5.2% for individuals who exclusively used home testing from January 1 to March 16, 2022 (12). Second, negative home tests were not reported to the CDCMS. However, while negative laboratory-confirmed tests were reported to the ECLRS, they were not part of this analysis. Thus, the impact is minimal. Third, deterministic database matches may have excluded potential matches when identifiers were not identical, such as when dates of birth were incorrect or names were misspelled or listed under maiden names instead of married names. Fourth, this analysis was limited to cases with completed interviews; those with complete interviews (those willing to participate in disease surveillance) maybe different. Fifth, this analysis included the period when universal COVID-19 case investigation and contact tracing were no longer required (34). Therefore, this study was based solely on COVID-19 case investigations conducted during the study period. Sixth, while this study identified demographic and health differences in COVID-19 test usage, it did not examine the social determinants of test usage, which warrants further investigation. Seventh, it is worth noting that closures and shutdowns, even though there were no statewide closures or shutdowns in effect during this time, may have impacted the choice of test types (35). Finally, these findings cannot be applied to other states, as they were based solely on data from NYS, excluding NYC. Furthermore, the COVID-19 cases and death rates in NYS, excluding NYC, were lower compared to those in NYC (36, 37).

These findings indicated that individuals who were excluded from official metrics were less likely to be hospitalized and were those with lower potential for severe disease, as measured by factors such as age, vaccination status, and underlying conditions.

## Data availability statement

The data presented in this study are available upon reasonable request from the corresponding author. Requests to access these datasets should be directed to VD, vajeera.dorabawila@health.ny.gov.

## Ethics statement

The studies involving human participants were reviewed and approved by the New York State Department of Health Institutional Review Board (IRB) which determined this surveillance activity to be necessary for public health work and therefore did not require IRB review. Written informed consent for participation was not required for this study in accordance with the national legislation and the institutional requirements.

## Author contributions

VD conceptualized and drafted the manuscript. VD, VB, NR, and RH contributed to file creation and analysis. VD, DH, AR, and EL contributed to critical revisions of the manuscript. VD, DH, JS, and ER contributed to the concept and design. All authors reviewed and approved the manuscript draft and ensured its accuracy and integrity before submission.
